# Reels to Remembrance: Attention Partially Mediates the Relationship Between Short-Form Video Addiction and Memory Function Among Youth

**DOI:** 10.3390/healthcare13030252

**Published:** 2025-01-26

**Authors:** Obada Al-Leimon, Wei Pan, Abdul-Raheem Jaber, Ahmad Al-Leimon, Abdel Rahman Jaber, Mohammad Aljahalin, Latefa Ali Dardas

**Affiliations:** 1 School of Medicine, The University of Jordan, Amman 11942, Jordan; 2 School of Nursing, Duke University, Durham, NC 27710, USA; 3 School of Nursing, The University of Jordan, Amman 11942, Jordan

**Keywords:** short-form video, TikTok, internet addiction, memory, attention, cognitive functioning

## Abstract

**Background and Purpose:** The proliferation of short-form video content on social media platforms has led to increased user engagement but also raised concerns about potential addictive behaviors and cognitive consequences, particularly among youth. This study explored the prevalence of short-form video addiction (SVA) among Jordanian youth, its correlates, and its impact on attention and memory function, with an emphasis on understanding the mediating and moderating role of attention in the relationship between SVA and memory. **Methods**: Utilizing a cross-sectional survey design, data were collected from 1029 university students across 25 higher-education institutions in Jordan. **Results**: Half of the participants exhibited moderate to high levels of SVA. The findings indicated a significant increase in SVA scores among female students (*p* = 0.003), those of a younger age (*p* = 0.045), those with lower GPAs (*p* = 0.013), and those who dedicated fewer hours to study (*p* = 0.006). Notably, there was a significant and large correlation between SVA scores and students’ perceptions of user-generated content (*p* < 0.001). Attention partially mediated the relationship between SVA and memory function with excellent model fit indices (χ^2^(12) = 14.11, *p* = 0.05, RMSEA = 0.03, GFI = 0.99, IFI = 0.99, TLI = 0.98, CFI = 0.99). However, attention did not moderate this relationship, suggesting that the impact of SVA on memory is consistent across varying levels of attention. **Discussion**: The findings underscore the significant engagement of Jordanian youth with short-form video content and the potential cognitive risks associated with SVA. Interventions to manage attention could mitigate the adverse effects of SVA on cognitive functions. This study calls for a comprehensive approach to address SVA among youth, including the development of digital literacy programs, mental health support services, and policy interventions that promote a balanced digital ecosystem and responsible media consumption.

## 1. Introduction

Short-form video is a novel type of user-generated content (UGC) in social media that has grown exponentially in popularity, engaging millions of users worldwide. Initially popularized by platforms such as TikTok, this format has increasingly been adopted by major social media spaces, including Facebook, Instagram, YouTube, X (formerly Twitter), and Snapchat, among others. Short-form video platforms feature a vertical scrolling feed that enables users to swipe or scroll up and down to navigate between videos. As they scroll, new videos are seamlessly loaded, allowing users to watch content in a continuous sequential manner. These videos typically range in length from a few seconds to a few minutes.

While the problematic use of and addiction to social media have been extensively studied over the past years [1,2,3,4], short-form video platforms may pose an even greater risk of addiction due to their inherent unique features. Their short video durations make binge-watching effortless, as users can quickly consume multiple videos in a short time. Hyper-personalized algorithms for recommending videos to users, based on their activity within the app, play a critical role in enhancing the immersive experience of these platforms [5,6,7]. Additionally, these platforms encourage active user participation, such as creating and sharing short videos and playback videos to trending songs (lip-sync videos) [8] and engaging in “challenges”. These challenges often involve creating and sharing videos that follow a specific theme, dance routine, or creative concept [9,10]. The competitive and viral nature of challenges encourages repeated interactions with the platform as users strive to participate, gain social validation, or achieve trending status. This repeated engagement, coupled with the platform’s algorithmic reinforcement (e.g., likes, comments, and shares), contributes to prolonged screen time and excessive usage, both of which are hallmarks of addictive behaviors [2]. These factors can also make users more likely to experience feelings of withdrawal when they are not using the platforms. The more intense the feelings of withdrawal, the more likely users are to increase their use of short-form videos [2,3,4].

Despite the continuous results highlighting the negative impact of digital media on psychosocial and cognitive health, a study published in 2023 [11] highlighted a significant dearth of research focusing on the effect of addiction to short-form videos on users’ cognitive functions, such as attention and memory. Existing studies have predominantly focused on broader digital behaviors, such as addiction to the internet [12,13], Facebook [14], microblogs [15], mobile phones [16], and video games [17]. These studies suggest a link between internet addiction and attention, indicating that higher frequencies of internet use are correlated with reduced attention [18,19,20] and that addiction to internet use might be even associated with limited growth in brain regions associated with attention [21,22]. Hartanto et al. [23] expanded on these findings, offering critical insights into how excessive media use adversely affects executive attention, underscoring the broader cognitive consequences of prolonged digital engagement. Short-form videos, however, present a unique case. Their rapid, algorithmically curated delivery is specifically designed to maximize user engagement, distinguishing them from other forms of digital media. This raises concerns about their potential to negatively impact attentional abilities, which in turn may compromise memory processes.

Theoretically speaking, attention and memory are closely interlinked within human cognition. Attention acts as a selective filter, prioritizing sensory information for short-term storage and subsequent processing. This limited-capacity gateway determines what enters memory and potentially becomes a lasting recollection. Encoding, the process of consolidating information into memory, heavily relies on focused attention [24,25]. Based on this theoretical foundation, we propose that addiction to short-form videos may impair attention, thereby compromising memory function.

The specific aims of this study were to (1) explore the prevalence and pattern of SVA among youth, (2) explore the correlates of SVA, and (3) test attention levels as a potential mediator or moderator in the relationship between SVA and memory function. We aim to explore these questions in a sample of Arab youth for two key reasons. First, youth are markedly susceptible to the effects of short-form video applications. Drawn to their entertainment and relaxation-promoting features, they often use these platforms as coping mechanisms to manage the stresses and challenges of daily life [26,27]. This behavior has been linked to a decline in academic motivation, evident in reduced enthusiasm for learning and disengagement in classroom settings [28]. Second, Arab youth particularly are prolific users of social media platforms and are simultaneously recognized as a group at heightened risk for various mental health issues, including depression and suicidal tendencies [29,30].

This study addresses critical gaps in the existing literature as it explores the cognitive implications of SFV addiction, with a focus on attention and memory function. Unlike prior research that broadly examines internet addiction, our study uniquely concentrates on SFV platforms like TikTok and Instagram, which possess distinct features that may amplify their addictive potential and cognitive impacts. Additionally, this research expands beyond attention to investigate the underexplored impact of SFV addiction on memory function, providing a more comprehensive understanding of its broader cognitive effects. This study focuses on Arab youth, a highly engaged yet underrepresented population in global studies.

## 2. Methods

### 2.1. Design

We used a national cross-sectional survey structured based on the Joanna Briggs Institute critical appraisal checklist for studies reporting prevalence data [31]. This study was conducted in Jordan, an Arab country recognized for having one of the youngest populations globally, with a significant portion (63%) of its population being under the age of 30 [32]. Data were collected from 25 higher-education institutions across Jordan, strategically selected to represent the diverse geographical regions of the country: urban, suburban, and rural. Our selection methodology employed a weighted sampling technique, such that we assigned weights based on the distribution of higher-education institutions across Jordan’s geographic regions (urban, suburban, and rural) to ensure representative sampling. Institutions in densely populated areas received higher weights due to their larger student populations, while those in less populated areas were proportionally weighted to reflect their contribution to the overall youth population. The population densities and enrollment data used to determine these weights were provided by the Ministry of Higher Education (2021). The data collection process was completed between 30 December 2022 and 28 February 2023.

### 2.2. Measurements

#### 2.2.1. Short-Form Video Addiction Scale

Short-form video addiction was measured using Mu Honglei et al.’s scale [26]. It consists of items that address unsuccessful attempts to reduce short-form video (SFV) app usage, difficulties in focusing on studies due to SFV apps, the impact of SFV apps on social life, and feelings of agitation when not using SFV apps. Participants rated their agreement with each item on a 5-point Likert scale. The total score, calculated by summing the responses, indicated the level of SFV addiction, with higher scores representing greater addiction levels. In this study, the Cornbach’s α was 0.73.

#### 2.2.2. The UGC Perception Scale

This study utilized the UGC perception scale to evaluate how youth using short-form video platforms experience immersion. The scale consisted of five statements (e.g., “How much do short-form video platforms impact your daily life?”) measured on a five-point rating system, with higher scores representing more immersive feelings [33]. The reliability of the scale has been supported [34]. In this study, the Cornbach’s α was 0.72.

#### 2.2.3. The Prospective and Retrospective Memory Questionnaire

To evaluate memory function among the study participants, the prospective and retrospective memory scale was utilized [35]. This scale allowed participants to self-report the frequency with which they experienced various types of memory errors using a 5-point Likert-type response format: “Very Often”, “Quite Often”, “Sometimes”, “Rarely”, and “Never”. The scale consists of eight distinct factors, each reflecting different aspects of memory, as follows: prospective short-term self-cued, prospective short-term environmentally cued, prospective long-term self-cued, prospective long-term environmentally cued, retrospective short-term self-cued, retrospective short-term environmentally cued, retrospective long-term self-cued, and retrospective long-term environmentally cued. The scale has been used in previous research and showed strong psychometric properties [36]. In this study, the Cornbach’s α was 0.92.

#### 2.2.4. Attention Control Scale

The Attention Control Scale (ATTC) [37] was used to assess participants’ attention abilities. The scale consists of two subdomains: attention focusing, the ability to maintain attention on a single task or stimulus for a prolonged period, and attention shifting, the capacity to adjust one’s focus between different tasks or stimuli effectively. The ATTC encompasses a total of 20 items, each of which is rated on a four-point Likert scale, ranging from 1 (almost never) to 4 (always). The scale’s use has been supported among youth [38]. In this study, the Cornbach’s α was 0.82.

All tools were translated into the Arabic language following the procedures outlined by Chapman and Carter (1979) [39]. We also collected data on participants’ characteristics, including their educational institutions, year of study, major, GPA, average daily hours spent on smartphones and short-form video platforms, average daily studying hours, physical activity levels, and the presence of prior or current mental health or general health issues.

### 2.3. Data Analysis

Out of 1051 students who returned the questionnaire, 1029 responses were considered valid for further analysis. The rate of missing data was only 2.1%, which was less than 5%. Thus, following the rule of thumb in the literature [40], no missing data imputation was necessary in this case, and the 1029 completed cases were included in the data analysis. All the analyses were conducted using IBM SPSS Statistics 29.0.1 [41] and AMOS 29.0.0 [42]. Descriptive statistics were used to explore the prevalence of SVA scores among Jordanian youth. Testing attention as a potential mediator in the relationship between SVA and memory was estimated and interpreted using structural equation modeling (SEM), whereas the moderation analysis was conducted using multigroup SEM to compare model fit between unconstrained and constrained models. Both the mediation and moderation relations among the variables in the conceptual models were examined while controlling for participant background characteristics. These characteristics were selected using a linear model predicting SVA from multiple potential predictors based on previous research results. The SEM model included 33 parameters (16 path coefficients, 8 covariances, and 9 variances). Based on the rule of thumb in the literature (i.e., number of parameters × 20) [43], 660 participants were required to obtain robust estimates. Thus, this study with 1029 participants had sufficient power to produce stable parameter estimates and model fit indices.

## 3. Results

Out of 1051 students who returned the questionnaire, 1029 responses were considered valid for further analysis. Within this valid dataset, 31% were males, 56% were in their first two years of study, 45.2% were enrolled in medical sciences, 20% were enrolled in engineering and technology, and 21% were enrolled in arts and humanities. In terms of academic performance, the majority (43%) had a very good GPA. About 13% of the students reported having a medical condition, with 3.8% mentioning a mental health condition. The predominance of female participants in our sample reflects the demographic reality of university students in Jordan, where females constitute the majority, as documented by the Ministry of Higher Education and Scientific Research [44]. Similarly, the lower representation of students in their fifth or sixth years is attributed to the typical four-year duration of most academic programs, with extended years being characteristic of a few fields such as medicine. Additionally, the greater representation of medical sciences aligns with the larger enrollment numbers in these disciplines compared to others [44]. We inquired whether respondents had sought psychological help and examined their sources of support. Among those who sought help, 43.4% (202 respondents) turned to family, while 45.8% (213 respondents) sought assistance from friends. A smaller proportion, 4.7% (22 respondents), reached out to university faculty, and 6.0% (28 respondents) relied on religious leaders for psychological support. In total, 465 respondents reported their sources of psychological help. Table 1 details the participants’ characteristics.

**Figure 1:**
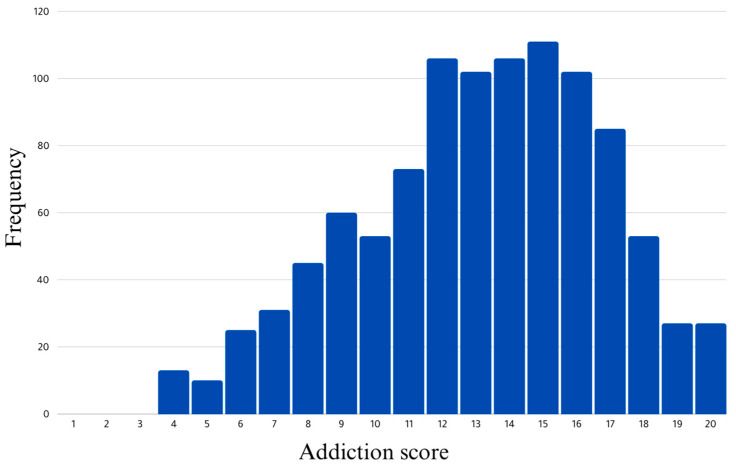
Distribution of short-form video addiction scores.

**Figure 2:**
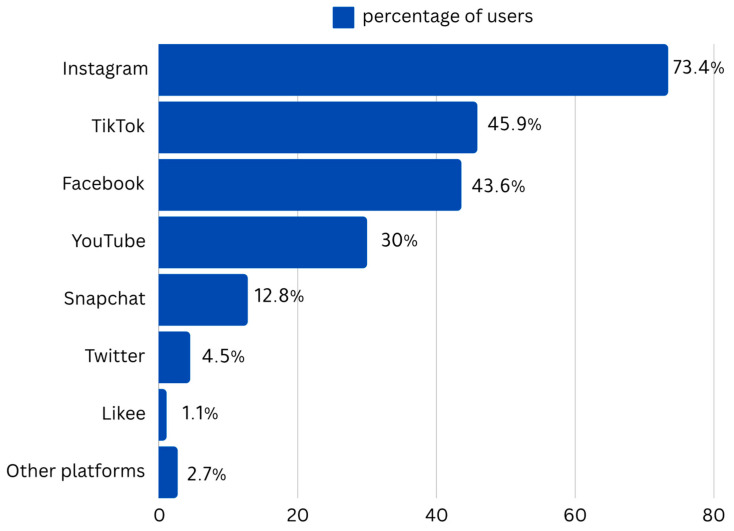
Percentage of users of different social media platforms.

The mean SVA score was 13.15 (±3.61), ranging from 4 to 20 (Figure 1). Of the total participants, 50% had SVA scores above 13, while 25% had SVA scores above 16. The platforms that were most frequently used for short-form video views were Instagram (73.4%), TikTok (45.9%), and Facebook (43.6%). Figure 2 displays all other platforms. Almost 18.8% of the participants used their smartphones for more than 8 hours each day. When asked about their perception of the TikTok ban imposed by the Jordanian government in December 2022, 15% of the participants expressed feeling annoyed, with an additional (6.5%) reporting extreme annoyance.

This study also identified correlates of SVA among youth, including academic major, gender, year of study, GPA, average study hours, psychiatric history, history of psychological assistance, and perceptions of UGC. The model explained 48% of the variance in SVA scores. The findings indicated a significant increase in SVA scores among female students, those of a younger age, those with lower GPAs, and those who dedicated fewer hours to studying and engaging in sports activities. Notably, there was a significant and large correlation between SVA scores and students’ perceptions of UGC (Table 2).

**Figure 3:**
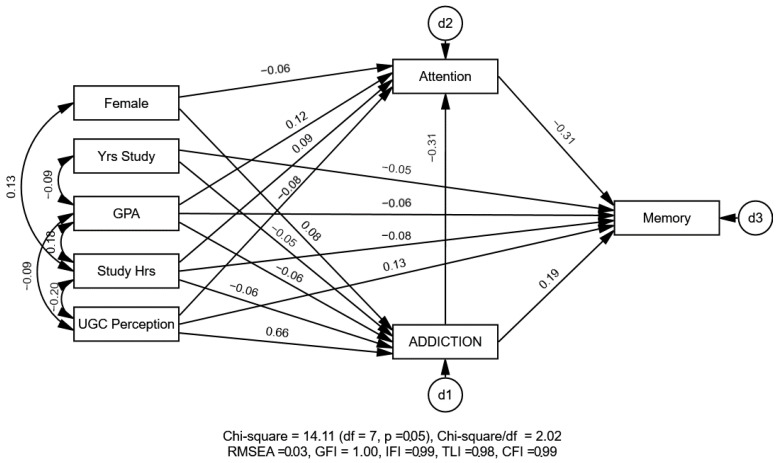
Attention partially mediates the relationship between short-form video addiction and memory. All the path coefficients were standardized and significant at the 0.05 level.

The mediation analysis tested attention as a potential mediator in the relationship between SVA and memory. Variables that were significant predictors for SVA scores in the linear model with *p* < 0.10 (Table 2) were selected as covariates in the mediation model. The results revealed that attention partially mediates the relationship between SVA and memory with excellent model fit indices (χ^2^(12) = 14.11, *p* = 0.05, RMSEA = 0.03, GFI = 0.99, IFI = 0.99, TLI = 0.98, CFI = 0.99). Figure 3 depicts both a significant direct effect for addiction scores on memory scores (γ = 0.19, *p* < 0.001) and a significant indirect effect for addiction on memory through attention (α × β = (−0.31) × (−0.31) = 0.10, *p* < 0.001).

**Figure 4:**
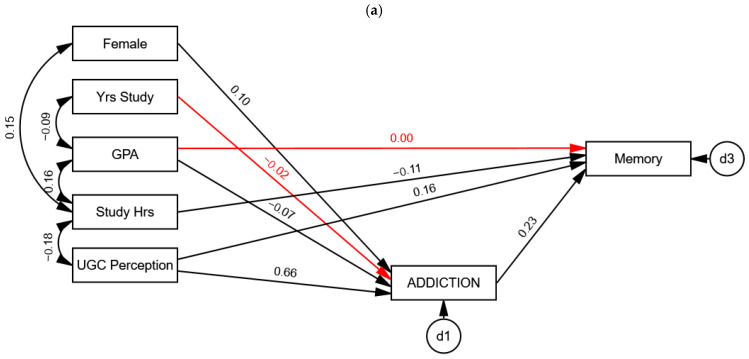

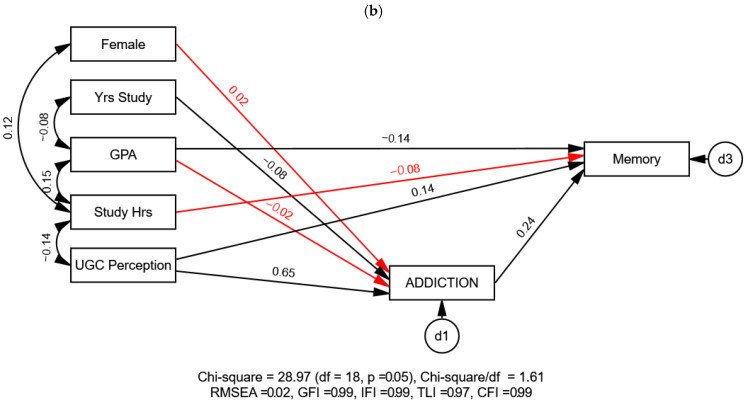
Attention did not moderate the relationship between short-form video addiction and memory. All the path coefficients were standardized. The black path coefficients were significant at the 0.05 level, and the red path coefficients were nonsignificant. (**a**) High attention (n = 536); (**b**) low attention (n = 493).

Testing attention as a potential moderator in the relationship between SVA and memory was analyzed using multigroup SEM, comparing the model fit between unconstrained and constrained models. Table 3 shows that the unconstrained model did not fit the data significantly better than the constrained model (Δχ^2^ = 11.00, Δ*df* = 8, *p* = 0.201), indicating that attention did not moderate the relationship between SVA and memory (SVA → memory: β_Hight Attention_ = 0.23, *p* < 0.001 and β_Low Attention_ = 0.24, *p* < 0.001). In other words, the relationship between SVA and memory was not different between students with high attention (score ≥ 49, the median value) and those with less attention (score < 49), as shown in Figure 4.

## 4. Discussion

The prevalent use of short-form videos, particularly among the youth, raises significant concerns regarding the potential negative consequences on their health and well-being. Given Jordan’s youthful population and the unprecedented popularity of short-form videos, this research aims to shed light on the prevalence and patterns of SVA, explore its correlates, and examine the potential mediating and moderating roles of attention in the relationship between SVA and cognitive functions, primarily memory.

The analysis of SVA levels among youth revealed that half of the participants had moderate to high engagement with short-form video content. This engagement is further underscored by the substantial daily smartphone usage reported by nearly a fifth of the participants. While comparing our results on a global scale is essential to understanding their relevance, such comparisons pose inherent challenges, primarily due to the limited availability of studies on SVA outside specific regions like China and South Korea. Despite these limitations, our analysis attempts to draw parallels and highlight differences by examining the available literature. The prevalence and patterns of SVA consumption observed in our study align with a study from China [26], which highlighted that a considerable proportion of adolescents (43%) spend one to two hours daily on short-form videos, with some (3.7%) even exceeding six hours per day, suggesting a deep engagement akin to our findings of moderate to high SVA addiction. Research from South Korea [45] found that a large segment of young adults aged 18–24 (40.15%) spends around two hours daily on short-form videos. Another study from China [46] reported that about 50% of the participants viewed short-form videos between one and three hours daily, closely mirroring our observations of substantial daily short-form video usage.

Predictive analysis identified several risk and protective factors influencing SVA scores. Particularly, female students and those with lower academic achievement showed higher SVA scores, pointing to potential implications for academic support and counseling services within educational institutions. However, it is important to acknowledge that the findings are based on a cross-sectional design, which restricts our ability to infer temporality or causal relationships between the variables studied. Longitudinal studies are needed to understand the directionality of these associations better. Further, addressing the relationship between SVA, attention, and memory function is inherently complex due to the dynamic interplay of social and behavioral factors in a rapidly evolving technological landscape. While our findings highlight these associations, we acknowledge the challenges of interpreting such interactions within a public health framework. Future research should consider stricter controls on social and behavioral variability to further elucidate these relationships and inform effective interventions for youth in an increasingly interconnected and technology-driven world.

Our moderation analysis, showing a lack of significant differences in the relationship across varying levels of attention, suggests that the impact of SVA on memory function is consistently present, regardless of an individual’s baseline attention capacity. This consistency across attention levels emphasizes the inherent risk posed by high SVA scores to cognitive health, reinforcing the need for comprehensive strategies to address this emerging challenge. On the other hand, the partial mediation by attention in the relationship between SVA and memory highlights the importance of attention management in mitigating potential adverse effects on memory.

These findings highlight opportunities for intervention, particularly through the development of digital literacy programs aimed at fostering healthier consumption habits among students. Addressing digital media addiction requires a multidisciplinary approach that combines insights from educational policy, mental health support, and digital literacy education. Such efforts can help create a balanced and healthy digital environment for young adults, but they are most effective when supported by government-level initiatives. However, the ethical implications of internet governance models play a critical role in managing digital addiction. For example, China’s government has implemented stringent regulations on content and screen time for children and adolescents to safeguard their well-being while promoting a productive digital learning environment [47]. These measures include mandatory restrictions, such as Douyin’s “teenager mode”, which limits screen time for users under 14 to 40 min per day and restricts access to specific content. In contrast, TikTok, which operates in countries like the United States and European Union, introduced a 60-min daily screen-time limit for users under 18 and optional parental controls. However, these measures are less enforceable, as users can often bypass restrictions by falsifying their age [47].

The Chinese governance model demonstrates the effectiveness of strict regulations in curbing excessive digital media use, but it also raises concerns about privacy and freedom of speech. Technologies like facial recognition and government-controlled content moderation have drawn criticism for their potential to infringe on individual rights. Conversely, governance models in the U.S. and EU prioritize data protection and individual freedoms, making it challenging to implement equally robust measures.

In Jordan, the government has taken a more restrictive approach by banning TikTok in response to concerns over content that violates community standards. However, many users circumvent this ban by utilizing virtual private networks (VPNs) to access restricted websites, routing their internet connection through servers in countries where such content is available. This highlights the ongoing tension between enforcing content restrictions and users’ ability to bypass them, further complicating efforts to regulate digital media effectively [48]. A key ethical dilemma remains: how to balance effective interventions with safeguarding privacy and personal rights. Future policies must address these challenges through evidence-based solutions that account for cultural, ethical, and political contexts.

## 5. Conclusions

Our research underscores the widespread engagement with short-form videos content among Jordanian youth, with a significant portion exhibiting behaviors indicative of addiction. This addiction is characterized not only by the duration of engagement but also by its impact on cognitive functions, particularly attention and memory. We advocate for a comprehensive approach to tackle SVA among youth in Jordan and globally. This approach should include the development of digital literacy programs, mental health support services, and policy interventions that promote balanced and responsible media consumption. Collaborative efforts among educators, policymakers, mental health professionals, and the tech industry are essential to foster a healthy digital ecosystem that supports the well-being and cognitive development of young individuals.

## Figures and Tables

**Table 1:** Demographic data of study participants (N = 1029).

Characteristic	Variables	n	%
**Gender**	Male	319	31.0
	Female	710	69.0
**Major**	Engineering and technology	208	20.2
	Medical sciences	465	45.2
	Applied sciences	68	6.6
	Arts and humanities	211	20.5
	Business and management	77	7.5
	Total	1029	100.0
**Year of Study**			
	First year	270	26.2
	Second year	301	29.3
	Third year	204	19.8
	Fourth year	137	13.3
	Fifth year	97	9.4
	Sixth year	20	1.9
**GPA**	Weak	12	1.2
	Fair	39	3.8
	Good	248	24.1
	Very good	441	42.9
	Excellent	289	28.1
**Study hours, average**	<2	279	27.1
	2–3	278	27.0
	4–5	267	25.9
	6–8	135	13.1
	>8	70	6.8
**Phone device usage hours, average**	<2	95	9.2
	2–3	283	27.5
	4–5	266	25.9
	6–8	192	18.7
	>8	193	18.8

**Table 2:** Predictors of short-form video addiction (N = 1029).

Variable	df	Mean Square	F	*p* Value
Study majors (5 categories)	4	9.51	1.39	0.237
Female (vs. male)	1	62.43	9.09	0.003
Year of study	1	27.74	4.04	0.045
GPA	1	42.31	6.16	0.013
Study hours	1	52.69	7.67	0.006
Psychiatric diagnosis (yes/no)	1	3.71	0.54	0.463
Receiving psychological help (yes/no)	1	4.63	0.67	0.412
UGC perception	1	5260.99	766.12	<0.001

R-squared = 0.481 (adjusted R-squared = 0.474).

**Table 3:** Unconstrained and constrained models for attention as a potential moderator in the relationship between short-form video addiction and memory.

Model	χ^2^	*df*	*p*	GFI	IFI	TLI	CFI	RMSEA	Model Comparison		
Unconstrained	28.97	18	0.049	0.99	0.99	0.97	0.99	0.02	Δχ^2^	Δ*df*	*p*
Constrained	39.98	26	0.039	0.99	0.98	0.97	0.98	0.02	11.00	8	0.201

## Data Availability

The data of this study are available from the corresponding author upon request.

## References

[1] Hou Y., Xiong D., Jiang T., Song L., Wang Q. (2019). Social Media Addiction: Its Impact, Mediation, and Intervention. Cyberpsychol. J. Psychosoc. Res. Cyberspace.

[2] Shannon H., Bush K., Villeneuve P.J., Hellemans K.G., Guimond S. (2022). Problematic social media use in adolescents and young adults: Systematic review and meta-analysis. JMIR Ment. Health.

[3] Marino C., Canale N., Melodia F., Spada M.M., Vieno A. (2021). The overlap between problematic smartphone use and problematic social media use: A systematic review. Curr. Addict. Rep..

[4] Musetti A., Manari T., Billieux J., Starcevic V., Schimmenti A. (2022). Problematic social networking sites use and attachment: A systematic review. Comput. Hum. Behav..

[5] Feldkamp J. (2021). The Rise of TikTok: The Evolution of a Social Media Platform During COVID-19.

[6] Qin Y., Omar B., Musetti A. (2022). The Addiction Behavior of Short-Form Video App TikTok: The Information Quality and System Quality Perspective. Front. Psychol..

[7] Pelet J.É., Ettis S., Cowart K. (2017). Optimal Experience of Flow Enhanced by Telepresence: Evidence from Social Media Use. Inf. Manag..

[8] Montag C., Yang H., Elhai J.D. (2021). On the Psychology of TikTok Use: A First Glimpse From Empirical Findings. Front. Public Health.

[9] Social Media Behavior Report-YPulse. https://www.ypulse.com/report/2023/02/22/social-media-behavior-report-3/.

[10] Ahlse J., Nilsson F., Sandström N. (2020). It’s Time to TikTok: Exploring Generation Z’s Motivations to Participate in #Challenges. https://www.diva-portal.org/smash/record.jsf?pid=diva2%3A1434091&dswid=-3392.

[11] Tian X., Bi X., Chen H. (2023). How Short-Form Video Features Influence Addiction Behavior? Empirical Research from the Opponent Process Theory Perspective. Inf. Technol. People.

[12] Marin M.G., Nuñez X., de Almeida R.M.M. (2021). Internet Addiction and Attention in Adolescents: A Systematic Review. Cyberpsychol. Behav. Soc. Netw..

[13] Zhang M.W.B., Lim R.B.C., Lee C., Ho R.C.M. (2018). Prevalence of Internet Addiction in Medical Students: A Meta-Analysis. Acad. Psychiatry.

[14] Hussain Z., Simonovic B., Stupple E.J., Austin M. (2019). Using Eye Tracking to Explore Facebook Use and Associations with Facebook Addiction, Mental WellBeing, and Personality. Behav. Sci..

[15] Hou J., Huang Z., Li H., Liu M., Zhang W., Ma N., Yang L., Gu F., Liu Y., Jin S. (2014). Is the Excessive Use of Microblogs an Internet Addiction? Developing a Scale for Assessing the Excessive Use of Microblogs in Chinese College Students. PLoS ONE.

[16] Li Y., Li G., Liu L., Wu H. (2020). Correlations between Mobile Phone Addiction and Anxiety, Depression, Impulsivity, and Poor Sleep Quality among College Students: A Systematic Review and Meta-Analysis. J. Behav. Addict..

[17] Walia B., Kim J., Ijere I., Sanders S. (2022). Video Game Addictive Symptom Level, Use Intensity, and Hedonic Experience: Cross-Sectional Questionnaire Study. JMIR Serious Games.

[18] Chen Y., Li M., Guo F., Wang X. (2023). The Effect of Short-Form Video Addiction on Users’ Attention. Behav. Inf. Technol..

[19] Augner C., Vlasak T., Barth A. (2023). The Relationship between Problematic Internet Use and Attention Deficit, Hyperactivity and Impulsivity: A Meta-Analysis. J. Psychiatr. Res..

[20] Peng M., Chen X., Zhao Q., Zhou Z. (2018). Attentional Scope Is Reduced by Internet Use: A Behavior and ERP Study. PLoS ONE.

[21] Takeuchi H., Taki Y., Asano K., Asano M., Sassa Y., Yokota S., Kotozaki Y., Nouchi R., Kawashima R. (2018). Impact of Frequency of Internet Use on Development of Brain Structures and Verbal Intelligence: Longitudinal Analyses. Hum. Brain Mapp..

[22] Fischer M., Moscovitch M., Alain C. (2021). A Systematic Review and Meta-Analysis of Memory-Guided Attention: Frontal and Parietal Activation Suggests Involvement of Fronto-Parietal Networks. Wiley Interdiscip. Rev. Cogn. Sci..

[23] Hartanto A., Chua Y.J., Quek F.Y.X., Wong J., Ooi W.M., Majeed N.M. (2023). Problematic Smartphone Usage, Objective Smartphone Engagement, and Executive Functions: A Latent Variable Analysis. Atten. Percept. Psychophys..

[24] Fu Y., Guan C., Tam J., O’Donnell R.E., Shen M., Wyble B., Chen H. (2023). Attention with or without Working Memory: Mnemonic Reselection of Attended Information. Trends Cogn. Sci..

[25] Oberauer K. (2019). Working Memory and Attention—A Conceptual Analysis and Review. J. Cogn..

[26] Mu H., Jiang Q., Xu J., Chen S. (2022). Drivers and Consequences of Short-Form Video (SFV) Addiction amongst Adolescents in China: Stress-Coping Theory Perspective. Int. J. Environ. Res. Public Health.

[27] Liu Y., Ni X., Niu G. (2021). Perceived Stress and Short-Form Video Application Addiction: A Moderated Mediation Model. Front. Psychol..

[28] Ye J.-H., He Z., Yang X., Lee Y.-S., Nong W., Ye J.-N., Wang C.-L. (2023). Predicting the Learning Avoidance Motivation, Learning Commitment, and Silent Classroom Behavior of Chinese Vocational College Students Caused by Short Video Addiction. Healthcare.

[29] Dardas L.A. (2022). Depression and Suicide among Arab Adolescents: 7 Messages from Research in Jordan. J. Psychiatr. Ment. Health Nurs..

[30] Maalouf F.T., Alamiri B., Atweh S., Becker A.E., Cheour M., Darwish H., Ghandour L.A., Ghuloum S., Hamze M., Karam E. (2019). Mental Health Research in the Arab Region: Challenges and Call for Action. Lancet Psychiatry.

[31] Munn Z., MClinSc S.M., Lisy K., Riitano D., Tufanaru C. (2015). Methodological Guidance for Systematic Reviews of Observational Epidemiological Studies Reporting Prevalence and Cumulative Incidence Data. Int. J. Evid.-Based Healthc..

[32] Youth|UNICEF Jordan. https://www.unicef.org/jordan/youth.

[33] Lu L., Liu M., Ge B., Bai Z., Liu Z. (2022). Adolescent Addiction to Short Video Applications in the Mobile Internet Era. Front. Psychol..

[34] Rigby J.M., Gould S.J.J., Brumby D.P., Cox A.L. Development of a Questionnaire to Measure Immersion in Video Media: The Film IEQ. Proceedings of the 2019 ACM International Conference on Interactive Experiences for TV and Online Video, TVX 2019.

[35] Smith G., Del Sala S., Logie R.H., Maylor E.A. (2000). Prospective and Retrospective Memory in Normal Ageing and Dementia: A Questionnaire Study. Memory.

[36] Butt M.A. (2023). Out-of-Home Mobility: A Measure of Daily Cognition in Young Adults.

[37] Derryberry D., Reed M.A. (2002). Anxiety-Related Attentional Biases and Their Regulation by Attentional Control. J. Abnorm. Psychol..

[38] Xie J., Xu X., Zhang Y., Tan Y., Wu D., Shi M., Huang H. (2023). The Effect of Short-Form Video Addiction on Undergraduates’ Academic Procrastination: A Moderated Mediation Model. Front. Psychol..

[39] Chapman D.W., Carter J.F. (1979). Translation Procedures for the Cross Cultural Use of Measurement Instruments. Educ. Eval. Policy Anal..

[40] Dong Y., Peng C.-Y.J. (2013). Principled missing data methods for researchers. SpringerPlus.

[41] (2023). IBM SPSS Statistics for Windows.

[42] Arbuckle J.L. (2022). Amos for Windows.

[43] Kline R.B. (2015). Principles and Practice of Structural Equation Modeling.

[44] Ministry of Higher Education and Scientific Research Annual Monitoring and Evaluation Report. https://moe.gov.jo/sites/default/files/moe_me_report_english.pdf.

[45] Peng C., Lee J.-Y., Liu S. (2022). Psychological Phenomenon Analysis of Short Video Users’ Anxiety, Addiction and Subjective Well-Being. Int. J. Contents.

[46] Ye J.H., Wu Y.T., Wu Y.F., Chen M.Y., Ye J.N. (2022). Effects of Short Video Addiction on the Motivation and Well-Being of Chinese Vocational College Students. Front. Public Health.

[47] Yang Z. (2023). How China Takes Extreme Measures to Keep Teens off TikTok. MIT Technology Review.

[48] Zhuravskaya E., Petrova M., Enikolopov R. (2020). Political Effects of the Internet and Social Media. Annu. Rev. Econ..

